# Case Report: I feel like a mother to other babies: experiences and perspectives on bereavement and breastmilk donation from Vietnam

**DOI:** 10.3389/fgwh.2023.1198738

**Published:** 2023-11-13

**Authors:** Hoang Thi Tran, Tuan Thanh Nguyen, Oanh Thi Xuan Nguyen, Roger Mathisen, Tanya M. Cassidy

**Affiliations:** ^1^ Neonatal Unit and Human Milk Bank, Da Nang Hospital for Women and Children, Da Nang, Vietnam; ^2^ Department of Pediatrics, School of Medicine and Pharmacy, Da Nang University, Da Nang, Vietnam; ^3^ Alive & Thrive, Global Nutrition, FHI 360, Hanoi, Vietnam; ^4^ School of Nursing, Psychotherapy, and Community Health (SNPCH), Dublin City University (DCU), Dublin, Ireland

**Keywords:** anthropology, bereavement, breastfeeding, child health and nutrition, human milk, human milk banking, neonatal mortality, vulnerable babies

## Abstract

There is a growing recognition globally that care regarding lactation following a perinatal death needs to potentially offer the opportunity for maternal donation. This article discusses this experience and perspectives from a human milk bank (HMB) in Vietnam. This is a descriptive exploratory case study that has a long tradition in both the social and health sciences. Triangulated data collection involved a review of video data, interview data with the donor, and data review for the Da Nang HMB, a Center for Excellence in Breastfeeding. We found that although it is common for mothers in Vietnam to donate breastmilk to HMBs, it is less common for this to occur following perinatal loss. We offer a descriptive case study of the maternal loss of twins and a subsequent choice to donate for approximately 1 month to the Da Nang HMB, the first HMB in Vietnam. We discuss four reasons derived from this case regarding donation following perinatal loss. (1) A strong motivation to donate breastmilk when aware of the service, (2) donating breastmilk helped her deal with grief, (3) family members supported her through this tough time and supported her decision, and (4) health staff supported her decision. While human milk sharing (e.g., wet nursing) has been practiced in Vietnam, breastmilk donation from bereaved mothers has neither been discussed nor well-researched. Because maternal grief is complex and individual, deciding to donate breastmilk is a personal decision that needs to be supported, without creating guilt for those who do not wish to donate.

## 1. Introduction

UNICEF reports that children “face the highest risk of dying in their first month of life at an average global rate of 17.6 deaths per 1,000 live births in 2021, down by 54 percent from 37 deaths per 1,000 in 1990” (1). UNICEF also reports that these same rates have also dropped significantly in Vietnam, and report that the Vietnamese neonatal mortality rate in 2020 is now under 10 per 1,000 live births, as was indicated in a survey conducted by the General Statistics Office in Vietnam and supported by UNICEF (2). Despite these reductions, neonatal deaths still occur, the suffering of the families that experience the loss is present. Research indicates that parental, particularly maternal, grief is present and highly complicated, especially when considering physiological experiences (e.g., hormonal changes, breast engorgement, uterine contractions, and weight loss) associated with lactation, as well as emotional and psychological changes (e.g., grief, sadness, anger, and guilt) (3, 4). There is a growing global recognition that healthcare providers need to support maternal grieving paying attention to physiological changes which will be experienced following the death of infant(s), validating decisions regarding continuing lactation while recognizing that for some people donation can potentially help to alleviate the emotional pain associated with perinatal loss (5). In this article, we concentrate on a specific case from Vietnam. Throughout the discussion of this specific case following the death of twins, and maternal donation to the first human milk bank (HMB) in Vietnam, we present this case to help shed light on an under-researched part of the world regarding the topic of bereavement, lactation, and potential maternal milk donation.

### 1.1. Human milk donation is common across the world

The twenty-first century has seen an exponential expansion of HMBs around the world, with almost 800 services being recognized in approximately 70 countries around the world, including increasing numbers in low- and middle-income countries (6). The World Health Organization (WHO) has recommended the optimal use of donor human milk from an HMB in preference to commercial milk formula and especially for small or sick infants, including low- and middle-income countries (7, 8). The demand for donor milk is high for vulnerable infants (9, 10), even for full-term newborns (9). Previous studies have shown that prelacteal feeding, especially with commercial milk formula, is negatively associated with exclusive and continued breastfeeding (11–13). There is a need for the development of HMB guidance, further expansion of the HMB network, and the inclusion of HMB as a part of breastfeeding policy (6, 14).

### 1.2. Human milk donation among bereaved mothers is not well-studied

Recently we are seeing increased research on HMB donations following a perinatal loss (15–17), although there is a long tradition of looking at this topic in North America (18) and Ireland (19). In 2019 the non-profit PATH released “A Resource Toolkit for Establishing and Integrating Human Milk Bank Programs,” with its final chapter dedicated to how to engage bereaved mothers in HMB services. The authors suggested that this complex and sensitive issue should be discussed with global health considerations (20).

Until recently, this topic has not been well-researched, and in some cultures, it has not even been discussed (21). Research shows, however, that donating breastmilk can have advantages for families, including helping with grief by acquiring a donor identity (15), and providing structure around bereavement (16). Yet, donating under these circumstances is not a decision that every person may choose or be able to make, or be offered. For women who wish to become a donor but are physically unable to, further support must be provided to avoid an additional sense of loss as well as mental and emotional health complications. At the same time, we must also make sure that under these very emotional circumstances that a potential donor is in no way feeling coerced to donate. This form of donation must be to help the mother through her journey of grief, and therefore may not be a part of the journey for everyone.

#### 1.2.1. Purpose of study

This study was designed to offer a descriptive exploratory account of breastmilk donation after perinatal death in the first HMB in Vietnam. We described a rare case of a donor who after the death of her twins started donating her milk to the first HMB in Vietnam, an important internationally recognized center of breastfeeding excellence, and explain the reasons/motivations for her donation.

#### 1.2.2. Type of research

This is a qualitative exploratory descriptive case study (22, 23), which involves exploring a specific phenomenon (in this case bereavement and donation) using more than one method (documentary analysis, interview, and mini ethnography), to describe in detail a specific case of bereavement and breastmilk donation in Vietnam. We then used pragmatic ethnographic analysis, to unpeel the layers of meanings (24) of the bereaved mother, the HMB, and Vietnamese culture in general (25, 26).

#### 1.2.3. Research questions

Why did a mother start or continue donating her milk after her children passed away? What are the barriers for her to do so? What are the facilitating factors? What can a health worker do to support the woman's and her family's decision?

## 2. Methods

### 2.1. Conceptual model and theory

Our study is informed using a form of mini ethnographic data collection which has been linked by Fusch et al. (27) and has been used for teaching particularly in South Asia (28) and linking these educational aspects to case study designs (26, 29). Ethnography has its roots in both anthropological and sociological theories, and some have argued it “has long been synonymous with case studies, typically conceived of as grounded in the local and situated in specific, well-defined, and self-contained social contexts” (29, 30). Using a mini form of ethnography follows the tradition in health services of having shorter, more focused ethnographic data collection, while still framing analysis around an ethnographic epistemology. Ethnographers and qualitative researchers in general explore how individuals make sense of their social worlds and wish to further understand those worlds or cultures, we can employ pragmatic ethnographic frames, giving a detailed description and interpretation of the culture or social world. In this case, we are describing the maternal social world of bereavement and donation in Vietnam.

### 2.2. Setting and study context

The case study was conducted at Da Nang Hospital for Women and Children (the hospital), a facility with a reputation for excellence. The hospital is designated as one of the three World Health Organization's Centers of Excellence for newborn care in Vietnam (31). It is also a referral hospital responsible for technical support and supportive supervision of district hospitals in Da Nang and other provinces in central Vietnam. Each year, 15,000 births take place in the hospital, and the facility supports 420,000 outpatients as well as over 30,000 women and 65,000 children inpatients. Because of its solid foundation for maternal and newborn care, including maternity promotion, protection, and support, the hospital was selected as the site for the first HMB in Vietnam (14).

The HMB in Da Nang, established in February 2017 is the first HMB in Vietnam and the model for establishing and improving HMBs in Vietnam and other East and Southeast Asian countries (14). Onsite events, social media, televisions, posters, and leaflets help to raise awareness of the HMB. Some donors contact health workers, mostly from the HMB or neonatal units to express their interest in becoming breastmilk donors. Health staff also directly contact potential donors, mostly from neonatal units, to recruit them (10). Upon consent to be a human milk donor, all donors are tested for HIV, hepatitis B and C, and syphilis. They also complete screening questions regarding recent treatments (e.g., blood transfusion or blood products, tuberculosis or cancer, medications contraindicated for breastfeeding), vaccination within 4 weeks, and risky behaviors (e.g., smoking, alcohol drinking, drug use, unsafe sex). The staff equips donors with essential skills in hygienic practices, expression, and storage of donor breastmilk. Donors have the choice to use their breast pump or use or borrow hospital breast pumps if they do not prefer hand expression. For donors from the surrounding community, HMB staff visit their residences weekly to check in with families, ensure maternal milk safety, provide clean containers, and collect the donated milk (10). In addition, throughout the entire process, healthcare workers provide breastfeeding support, including psychological support, lactation assistance, and milk donation guidance. This support encompasses, but is not limited to, answering questions, addressing concerns, helping women overcome breastfeeding and donation challenges, and assisting in making decisions about ending donations.

As of April 2022, monitoring data showed an enormous impact—516 breastmilk donors together donated 9,777 liters of milk that was pasteurized to support and nourish 24,079 newborns.

### 2.3. Sampling

This case study, like many qualitative studies, involves purposive sampling and non-random sampling, which can support an in-depth study and give information-rich cases (32). Since the opening of the HMB in Da Nang there have been 516 donors, and three donated breastmilk after their children's death at the hospital. Two donors started donating when their newborns were under treatment at the neonatal unit. They continue donating for a few days after the deaths of their newborns. They returned to their home provinces, and thus stopped donating breastmilk. The remaining donor was the only one who started donating milk after her newborns passed away and continued doing so for about a month. Even though the case was from 2018, the hospital staff stayed in contact with her. Furthermore, she was featured in a national TV program. We contacted her to seek her permission to use her story for this write-up and she agreed. We also contacted and obtained permission to use the content from the TV program for our write-up, and had it translated into English with subtitles. This was then made available for the first time during a webinar hosted by the UK Association of Milk Banking (UKAMB) and Dublin City University (DCU) in November 2021 and is available on YouTube (33). The third coauthor supported the donor through the entire process and stayed connected with her after the last donation. This author was the focal contact with this woman for this article. Ethical approval was obtained for this case report from the Scientific and Ethics Board of the hospital and written informed consent and permission from the mother were obtained.

### 2.4. Data collection

We used three data collection sources for our mini-ethnographic case study, which supports a sense of triangulated confidence in our results.

First, we gathered data from the HMB regarding the social and cultural context around donation in Vietnam (9).

Second, we gathered information about this case. The mother donated in 2018 and participated in a documentary about the HMB. The original Vietnam Television Channel 1 (VTV1) documentary also aired in late 2018 (34). Staff had stayed in touch with the donor, and so we contacted her again when we were preparing this research to get her consent to be part of the study in late 2021 for the presentation and early 2022 for this case study. The VTV1 original documentary was clipped to include only the materials related to this donor and English subtitles were produced and presented at a webinar hosted by Dublin City University (DCU) School of Nursing, Psychotherapy, Community Health (SNPCH) and the UK Association of Milk Banking (UKAMB) (33).

We also engaged with the donor after that through contact with the health worker, who is the third co-author of this article. To prepare for this case study, in early 2022, the health worker contacted her again to seek her approval for her story to be used in this research article and sought additional information to help frame our discussion. This documentary and interview data were triangulated with a review of data from the HMB, to build a social and cultural mini-ethnographic context around donation in Vietnam.

### 2.5. Data analysis and presentation

Ethnographic data analysis is an iterative, spiral, and self-reflective process (35). All of the authors have been involved in the HMB since before it existed, as the collaboration occurred during a separate ethnographic study on donor human milk services which one of the authors conducted in the UK (21). In addition, one author conducted the donor interviews, and all authors reviewed the documentary as mini-ethnographic data, especially after English subtitles were available (35). Although a descriptive case study (22), our data was analyzed from an abductive ethnographic frame (36), which offers some explanatory discussions related to the details discussed in this case.

## 3. Results

### 3.1. A case study: donating breastmilk after bereavement

Ms. Hoa (pseudonym, even though she consented to using her real name) was 20 years old and working in a factory in Da Nang, Vietnam. In early 2018, she married her husband, and soon announced her first pregnancy. At her first trimester check-up, Ms. Hoa and her husband were thrilled to learn that she was expecting twins. However, 5 months into her pregnancy, the unimaginable happened—after feeling signs of labor in the night, she experienced a spontaneous membrane rupture. She was immediately rushed to the hospital, but her babies could not be saved. The unexpected loss left Ms. Hoa, as well as her family, grieving and devastated.

In the following days, while she was still in the hospital, Ms. Hoa experienced breast engorgement. Her doctor offered medications to stop her milk production, but Ms. Hoa had seen information about the HMB and contacted hospital staff to learn more about the process. Ms. Hoa was moved by the idea of becoming a breastmilk donor, thinking about the support and relief she could give to other mothers, as well as the vital, life-saving nutrition she could provide to vulnerable newborns. Over the next month, she donated a total of 5.3 L of breastmilk, which was used to support preterm and sick newborns. All the breastmilk Ms. Hoa donated met the strict safety standards set by the HMB.

The above story was told by Ms. Hoa and the HMB staff (the third author) and was featured on a National Television program (34).

In Vietnam, it is uncommon for bereaved mothers to donate breastmilk, and the option is rarely offered. This makes the donation from Ms. Hoa even more unique. Only three generous donors at the Da Nang HMB, out of 516 total donors over the past 5 years, have been bereaved mothers—including Ms. Hoa.

According to the Vietnam Ministry of Health, in the main catchment area of the hospital (Da Nang City, Quang Nam and Quang Ngai provinces), the number of neonatal deaths is approximately 84 out of 54,000 annually, translating to 420 deaths over 5 years at a neonatal mortality rate of 1.54 per 1,000 live births. Nationwide, 17,000 neonatal deaths (at a neonatal mortality rate of 11 per 1,000 live births) were recorded in 2018 (37). Thus, the potential number of donors like Ms. Hoa could be much higher.

### 3.2. Why Ms. Hoa donated after her loss

Ms. Hoa had a strong motivation to donate breastmilk. She said:

*“I did not take medications to stop my milk production—I wanted to bring my breastmilk to other babies, especially premature babies like the ones I lost, or babies who are sick or whose mothers can't produce breastmilk. I wanted to bring breastmilk to the babies who don't have access to it. As long as I can produce milk, I know I'm doing something to support other babies—I will keep on giving every drop until it naturally stops. I hope that each drop of love will help babies grow up strong.”*

For Ms. Hoa, donating breastmilk also helped her deal with her grief. She said:

*“Expressing breastmilk gave me the strength to continue with my life. My babies will not come back to me. But through donating the milk I made for the babies I lost, I feel like a mother to other babies.”*

Ms. Hoa had family members who supported her through this challenging time. The HMB staff observed:

*“Ms. Hoa and her family lived in a small house in a poor, suburban area of Da Nang City. They had only one old fridge to store food. When I explained the requirement of a clean fridge to store breastmilk, her mother-in-law, without hesitation, took out the food in their only fridge, cleaned it, and gave Ms. Hoa the space to store donor breastmilk.”*

HMB staff supported Ms. Hoa both physically and mentally and formed a close relationship that allowed her to donate milk during her hospital stay and after discharge. They helped her to channel her grief, as she missed her babies during milk expression. Later Ms. Hoa reflected: “the staff helped me understand that to give is to receive. Indeed, I received happiness in return. I got to provide for other babies, and then, I got pregnant again and have now two beautiful and healthy children.”

## 4. Discussion

### 4.1. The culture of human milk sharing in Vietnam

Human milk sharing (e.g., wet nursing), either paid or unpaid, has been practiced since ancient times in many countries in the world (38, 39). Wet nurse—“a woman who breastfeeds another's child”—was considered a popular, well-paid, and highly organized profession (38). Some countries, including France, required wet nurses to register at a municipal employment bureau and had laws to regulate their employment (38).

In Vietnam, nursing mothers were commonly hired by well-off families (e.g., in North Vietnam before 1954 and South Vietnam before 1975) in exchange for money, food, and lodging (40). However, public opinion considered wet nursing an evil of feudalism and colonialism, and exploitation of women, and the practice eventually became less commonplace (40). Similar exploitive features of wet nursing also led to its pejorative features in other countries around the world, contributing to the reason that many HMBs around the world do not have commercial payments associated with milk donations. Wet nursing, a woman breastfeeding another's child without payment, often occurs around the world and has been labeled by some as “cross nursing” to avoid the pejorative links to the term wet nursing (41).

HMBs are to provide human milk to small and sick infants while addressing the concern of transmitting diseases associated with wet nursing such as HIV, hepatitis B and C, and syphilis (14, 42). However, the donation of breastmilk to an HMB is a new concept in Vietnam. Based on our estimation, the current four active HMB in Vietnam only cover a catchment area of about seven percent of the total number of newborns throughout the country. Also, not all mothers have the capacity or are willing to donate their milk. In the last 5 years, only 516 mothers out of the 75,000 births became donors at the Da Nang HMB.

Mothers who have lost their babies typically do not start or continue donating their breastmilk. Of the three bereaved donors in Da Nang, Ms. Hoa is the only one who continued donating for an extended period. The other two donors donated only briefly after their children passed away. Potential reasons could include that donating reminds mothers of their loss. Mothers and other family members may also think that continuing to express breastmilk may prolong their grief.

It is worth noting that abortion, miscarriage, and surrogacy mothers also experience similar feelings of loss especially when the women have the capacity of lactating, although these experiences may be different in important ways as well (43, 44).

For Ms. Hoa and her family, the breastmilk donation helped to alleviate her sadness significantly. This “maternal generosity” and the communal nature of maternity underlies donor human milk services (21). The support from Ms. Hoa's mother-in-law and family members is also important given their influence in decision-making about maternal, infant, and young child nutrition (45, 46). However, due to the complicated and individual nature of grief, bereaved families should never be made to feel like they must or should donate.

In the 5.5 years of operation, HMB staff often do not actively approach mothers who have lost their babies for donation. First, the staff are afraid of touching the mothers' and families' sadness. However, donations can alleviate sadness as in this case study. We need to be incredibly careful if presenting this option that donors in no way feel a sense of coercion, but instead are aware that some people have found this helpful and that they were only being told in case they also found it helpful. Second, the volume of donor breastmilk received from other mothers is in surplus for the Da Nang HMB to be used by the hospital and hospitals in need in Da Nang and neighboring provinces (9).

### 4.2. What more do we need to learn?

Knowledge is limited to mothers' opinions on milk donation after losing their babies. For example, what would a mother do to her frozen stored milk? Why does a grieving mother start or continue donating her milk? Does the donation improve or aggravate their feelings of loss? What is the perception of recipients' families on donor milk from bereaved mothers? Are there cultural variations in bereavement and donation?

## 5. Conclusion

In this case study, we found that the bereaved mother donated milk for a month with the support of her family and health workers. The woman was determined to donate milk, and this act helped her alleviate her feelings of loss after losing two children.

Because maternal grief is complex (4) and individual, deciding to donate breastmilk is a personal decision that needs to be supported, without creating guilt for those who do not wish to donate. Health workers, including those working in the HMB, should make sure that mothers and other family members know that a donation is an option if they wish to do so. HMB networks around the world need to exchange information, experience, lessons learned, research findings, and appropriate policies to bolster learning on this topic. The HMB networks can also develop culturally appreciative guidelines to help shape the practice globally. With the world of online information and support from HMB staff, bereaved families may learn about practices and policies in their own and other countries to have informed, appropriate decisions.

## Key messages

• Few bereaved mothers donate breastmilk, and the phenomenon is not well understood.
• A case of a mother who became a breastmilk donor after her twins' death showed that she had a strong motivation to donate, felt eased with the donation, and had support from family members and health workers.
• Because maternal grief is complex and individual, deciding to donate breastmilk is a personal decision that needs to be supported.
• Health workers and human milk bank networks play an important role in sharing information, guiding, and supporting bereaved mothers with informed appropriate decisions.

## Data Availability

The original contributions presented in the study are included in the article/Supplementary Material, further inquiries can be directed to the corresponding author.
